# Out-of-pocket healthcare expenditure in Australia: trends, inequalities and the impact on household living standards in a high-income country with a universal health care system

**DOI:** 10.1186/s13561-019-0227-9

**Published:** 2019-03-11

**Authors:** Emily J. Callander, Haylee Fox, Daniel Lindsay

**Affiliations:** 1 School of Medicine, Griffith University – Gold Coast campus, G05 Room 2.24, Southport, Queensland 4125 Australia; 2 College of Public Health, Medical and Veterinary Science, James Cook University, Townsville, QLD 4810 Australia

**Keywords:** Out-of-pocket expenditure, Universal health care, Australia, Inequality, Household living standards, Poverty

## Abstract

**Background:**

Poor health increases the likelihood of experiencing poverty by reducing a person’s ability to work and imparting costs associated with receiving medical treatment. Universal health care is a means of protecting against the impoverishing impact of high healthcare costs. This study aims to document the recent trends in the amount paid by Australian households out-of-pocket for healthcare, identify any inequalities in the distribution of this expenditure, and to describe the impact that healthcare costs have on household living standards in a high-income country with a long established universal health care system. We undertook this analysis using a longitudinal, nationally representative dataset – the *Household Income and Labour Dynamics in Australia* Survey, using data collected annually from 2006 to 2014. Out of pocket payments covered those paid to health practitioners, for medication and in private health insurance premiums; catastrophic expenditure was defined as spending 10% or more of household income on healthcare.

**Results:**

Average total household expenditure on healthcare items remained relatively stable between 2006 and 2014 after adjusting for inflation, changing from $3133 to $3199. However, after adjusting for age, self-reported health status, and year, those in the lowest income group (decile one) had 15 times the odds (95% CI, 11.7–20.8) of having catastrophic health expenditure compared to those in the highest income group (decile ten). The percentage of people in income decile 2 and 3 who had catastrophic health expenditure also increased from 13% to 19% and 7% to 13% respectively.

**Conclusions:**

Ongoing monitoring of out of pocket healthcare expenditure is an essential part of assessing health system performance, even in countries with universal health care.

## Introduction

Poor health increases the likelihood of experiencing poverty by reducing a person’s ability to work and imparting costs associated with receiving medical treatment. Those who develop a chronic disease have a higher chance of leaving the workforce [29], and as such see a decline in their income as they lose the wages associated with paid employment [25]. This chain of events has been observed internationally [1, 24, 28] – as health, being a key form of human capital, universally effects a person’s *ability* to participate in employment [3]. Countries with a welfare system may provide an income safety net for those who are too ill to work, thus providing a [small] supplementary income stream in the form of transfer payments. None-the-less, multiple studies have shown that those who develop a chronic disease face an increased risk of falling into income poverty, even in High-Income Countries (HICs) with such welfare systems in place [4, 5, 7].

Poor health and the negative impact it can have on living standards is important for a number of reasons. Governments with welfare systems to support those who are too ill to work will see an increase in the number of transfer payments being made; the more people out of the labour force due to ill health reduces the revenue base from which governments can draw an income stream to finance these transfer payments; and from the individual perspective, declining income reduces the amount of disposable income available to finance access to healthcare. This illustrates the cross-portfolio issues associated with the health-living standards nexus; highlighting the far-reaching impacts that poor health can have on both the Government and individual’s financial capacity.

Poor health not only adversely affects people’s financial capacity due to withdrawal from the labour force; poor health can also affect financial capacity by increasing the amount of household expenditure on healthcare related items. Healthcare is more of a ‘necessary’ good, as opposed to a ‘discretionary’ good [17], with people often having little choice as to whether they access it or not. As such, increasing expenditure on healthcare has a similar effect to decreasing income: it reduces the amount of disposable income available to families to spend on other goods, such as food, education, transport and entertainment.

Universal health care means that all people have access to the health services they need without being exposed to financial hardship when doing so [8]. Poorer people within the population have the greatest need for health care as they are more likely to suffer from illness and disease [2]. Therefore, contributions should be based on ability to pay and health services should be allocated according to need, ensuring that high out-of-pocket healthcare costs are mitigated, and the associated impoverishing potential of poor health is reduced [8]. Australia has a universal health care system, Medicare, which was introduced in 1984. In response to the spiralling costs facing the Australian state to financing this system, ongoing health care reform has led to an interrogation of the amount being paid out-of-pocket by individuals [26]. Previous research in this area has looked at out-of-pocket expenditure at a single point in time [6, 35], or focused upon expenditure by a single sub-population [21, 32]. However, it has been noted that the basis of the Medicare system – to provide universal health care – is being undermined by increasing out-of-pocket costs [20].

Against this background, this study has three research questions:

1. What do Australians currently pay for household healthcare expenditure and how has this changed over time?
2. What proportion of individuals live in households that have ‘catastrophic healthcare expenditure’, and what is the distribution of catastrophic expenditure by income group?
3. How many additional people would be in income poverty when household income is adjusted for household healthcare expenditure?

The overall aim of this paper is to document the recent trends in the amount paid by Australians out of pocket for healthcare, identify any inequalities in the distribution of this expenditure, and to describe the impact that household healthcare costs have on living standards in a HIC with a long established UHC system. While internationally, much attention has been given to identifying catastrophic healthcare expenditure [30, 31, 34], to date this has been a relatively overlooked area within Australia. The studies that have been conducted to date have only looked at older Australians with chronic health conditions [21] or specific chronic health conditions [16], and none have looked at the population as a whole.

## Methods

### Australia’s healthcare system

Australia’s publically financed national universal health insurance scheme, Medicare, was introduced to promote equity by improving access and affordability of health services. Through Medicare, patients are able to access treatment in public hospitals free of charge, and receive subsidised out of hospital treatment. Patients are provided a rebate benefit for services that are utilised for out of hospital treatment. The rebate amount is based upon a proportion of a schedule of fees covering each type of service. For example, for a consultation with a General Practitioner lasting 20 min or more, the schedule fee in 2017 is $71.70, and the benefit is 100% of the schedule fee, or $71.70; a blood test associated with diabetes management has a schedule fee of $16.80 and the benefit is 75% of the schedule fee, or $12.80 [12]. While public hospitals are managed by the state, most out of hospital services are delivered by private providers. The actual amount charged by providers for services is set by the providers themselves, and these charges are not regulated, meaning that providers are able to set their fees above the schedule fee. Any difference between the price providers charge for a service and the rebate amount is paid by patients ‘out-of-pocket’. For illustration, if a provider charged $25.00 for a blood test associated with diabetes management, Medicare would provide a rebate of $12.80 (75% of the schedule fee), leaving the patient to pay $12.20. Medicare has policies designed to help protect patients from high out of pocket costs. Health Care Cards are provided to welfare recipients and low income earners, and entitles holders to pay a lower out of pocket fee for prescription medicines [13]. The ‘Medicare Safety Net’ and ‘Extended Medicare Safety Net’ Programs also provide higher rebates if an individual or family group reaches a certain amount of total expenditure on out of pocket fees within a calendar year. Any subsequent services or prescriptions will have a higher proportion subsidized for the rest of that calendar year [15]. Under the “Medicare Safety Net”, once the threshold is reached 100% of the schedule fee for all services is rebated; and under the “Extended Medicare Safety Net” 80% of the actual out-of-pocket fees are rebated. For Health Care Card holders the threshold of total expenditure that needs to be reached to receive the “Extended Medicare Safety Net” is lower [14].

### Dataset to be used for this study – HILDA

Microdata from waves 6 to 14 of the Household Income and Labour Dynamics in Australia (HILDA) Survey was utilised for this study. The HILDA survey is a longitudinal survey of private Australian households conducted annually since 2001, with release 14, containing data from waves 1 (2001) to wave 14 (conducted in 2014), the latest to be released at the time of writing this paper. The data are nationally representative of the Australian population living in private dwellings and aged 15 years and over. There were 6547 records of individuals aged 20 years and over in Wave 6 of the continuing sample HILDA survey, representing 10,381,000 people in the Australian population.

The survey sampling unit for Wave 1 from which the continuing sample is drawn was the household, with all members of the household being part of the sample that would be followed for the life of the survey. Household sampling was conducted in a three-stage approach. Initially, 488 Census Collection Districts (each containing 200 to 250 households) were selected. Within each district, 22 to 34 dwellings were then selected, and finally, up to three households within each dwelling were selected to be part of the sample [27]. The data is weighted to be representative of the Australian population and to account for any bias introduced through respondent attrition. The initial household cross-sectional weights in Wave 1 (upon which the weights in subsequent waves are dependent) were derived from the probability of selecting the household and were calibrated so that the weighted estimates match known benchmarks for the number of adults by the number of children and state by part of the state. The person-level weights were based on the household weights and then calibrated so that person weights match known benchmarks for sex by age, state by part of the state, state by labour force status, marital status and household composition. The longitudinal weights adjusted for attrition and were benchmarked against the characteristics of Wave 1. For a detailed description of HILDA weighting see Watson (2012). All dollar values in this study were adjusted to 2014 Australian dollars based upon Consumer Price Inflation (CPI) (2017) [23].

### Household healthcare expenditure

Wave 6 onwards in the HILDA survey asked respondents to estimate the amount the household spent annually on fees paid to:

- Health practitioners;

- Medicines, prescriptions, pharmaceuticals, alternative medicines; and.

- Private health insurance.

The reported amounts were recorded separately for each of the three categories. For the purpose of this study, the three groups were summed to create a total health care expenditure amount. All results are reported at the individual level, but for household expenditure.

### Household income

For this study, the total regular household income minus taxes was utilised. For the assessment of the distribution of healthcare expenditure, this measure of household income was equivalised using the OECD-modified (De [11]) equivalence scale. This accounted for the number of adults (aged 15 and over), and the number of children (aged 14 and under) living in the household.

### Catastrophic healthcare expenditure

Within Australia, there is no accepted threshold for what proportion of a household’s income makes expenditure on healthcare ‘catastrophic’. Therefore, will be using a threshold of 10%, based upon a previous study conducted within Australia, although other cut-offs have been used internationally [31]. Individuals who have 10% or more of their total regular household income minus taxes that are taken up by household healthcare expenditure are deemed to have ‘catastrophic’ healthcare expenditure [22].

### Impoverishing healthcare expenditure

Impoverishing healthcare expenditure is an expenditure that places a household’s income below the poverty line. The 50% of the median equivalised income poverty line was utilised, which is the accepted cut-off for poverty measurement in Australia [9] and differs from the 60% used in other countries [18]. The total amount of household expenditure on healthcare was subtracted from total regular household income minus taxes. This was then equivalised, again, using the OECD-modified (De [11]) equivalence scale.

### Statistical analysis

The initial descriptive analysis was undertaken to quantify the average out of pocket household expenditure on healthcare for each year between 2006 and 2014.

The proportion of people with catastrophic healthcare expenditure in each income decile was then identified. A generalised estimating equation model was then constructed to assess the odds of having catastrophic health care expenditure for those in different income decile. The model was adjusted for age, sex, self-assessed health status and year, with those in income decile ten used as the reference group.

A concentration index was constructed for each year between 2006 and 2014 to identify the cumulative proportion of people with catastrophic healthcare expenditure by cumulative proportion of the population, ranked by equivalised household income The concentration index (CI), and it’s associated 95% confidence intervals, were computed as follows:

$$ 2{\sigma}_R^2\left(\frac{y_i}{\mu}\right)=\alpha +\beta {R}_i+{\varepsilon}_i $$

Where *R*_*i*_ is the rank of each individual, ${\sigma}_R^2$ is the variance of *R*_*i*_, *y*_*i*_ is the catastrophic healthcare status of each individual (i = 1, 2, 3….N), *α* is the intercept, *ε*_*i*_ is the error terms, and *β* is the CI [19].

## Results

Table 1 shows the average amount of household expenditure on healthcare practitioners; medicines, pharmaceuticals, and alternative medicines; and private health insurance. Average total household expenditure on healthcare items has only slightly increased after adjusting for inflation between 2006 and 2014 from $3133 to $3199 (in 2014 dollars). This appears to be mostly driven by increases in private health insurance expenditure, which was on average $1242 in 2006 and increased steadily to $1557 in 2014. Average expenditure on healthcare practitioners decreased slightly between 2006 and 2014 from $1188 in 2006 to $1099 in 2014, and expenditure on medicines, pharmaceuticals, and alternative medicines remained somewhat constant.

**Table 1:** Mean total household out of pocket expenditure on healthcare costs; healthcare practitioners; medicines, pharmaceuticals, alternative medicines; and private health insurance, 2006–2014

Year	n	Mean	Std Error of Mean
Total healthcare expenditure			
2006	6265	3133	90
2007	6265	3065	50
2008	6324	3158	55
2009	6362	3073	52
2010	6390	3192	75
2011	6409	3162	61
2012	6421	3129	48
2013	6429	3198	53
2014	6429	3199	51
Expenditure on healthcare professionals			
2006	6265	1188	31
2007	6265	1195	29
2008	6324	1221	30
2009	6362	1152	32
2010	6390	1213	35
2011	6409	1241	46
2012	6421	1113	28
2013	6429	1122	31
2014	6429	1099	30
Expenditure on medicine, pharmaceuticals and alternate medicine			
2006	6265	703	77
2007	6265	607	20
2008	6324	590	18
2009	6362	589	14
2010	6390	560	10
2011	6409	563	11
2012	6421	570	12
2013	6429	575	14
2014	6429	544	12
Expenditure on private health insurance			
2006	6265	1242	22
2007	6265	1263	23
2008	6324	1348	29
2009	6362	1331	26
2010	6390	1418	59
2011	6409	1358	24
2012	6421	1447	25
2013	6429	1500	27
2014	6429	1557	28

Table 2 shows that the proportion of people with catastrophic healthcare expenditure decreases with income decile – with those in the lowest income decile having the highest percentage of people with catastrophic healthcare expenditure. The concentration index for the distribution of catastrophic expenditure was − 0.39 (95% CI: -0.43, − 0.34) in 2006, and increased to − 0.46 (95% CI: -0.50, − 0.42), showing an increase in the distribution of catastrophic healthcare expenditure towards those of lower income over time.

**Table 2:** Proportion of households with catastrophic healthcare expenditure by decile, 2006–2014

Income quintile	2006	2007	2008	2009	2010	2011	2012	2013	2014
1	31%	24%	25%	35%	25%	32%	33%	35%	31%
2	13%	17%	16%	20%	17%	21%	18%	19%	19%
3	7%	10%	15%	11%	17%	12%	16%	12%	13%
4	6%	9%	9%	6%	9%	10%	10%	7%	10%
5	8%	7%	8%	7%	9%	9%	9%	11%	9%
6	4%	5%	5%	3%	4%	5%	5%	3%	5%
7	4%	4%	6%	4%	4%	3%	5%	6%	5%
8	4%	3%	3%	3%	4%	2%	3%	4%	2%
9	3%	3%	4%	3%	2%	3%	3%	2%	2%
10	1%	2%	2%	1%	1%	2%	0%	3%	3%
*Concentration Index*	*−0.39 (−0.43, −0.34)*	*−0.40 (−0.45, −0.36)*	*−0.43 (−0.48, −0.39)*	*−0.39 (−0.43, −0.34)*	*−0.44 (−0.49, −0.40)*	*−0.48 (−0.52, −0.43)*	*− 0.53 (− 0.58, − 0.48)*	*− 0.46 (− 0.50, − 0.41)*	*−0.46 (− 0.50, − 0.42)*

Relative to those in the highest income decile, there was an increasing likelihood of having catastrophic healthcare expenditure with decreasing income decile. After adjusting for age, self-reported health status, and year, those in income decile one had 15.63 times the odds (95% CI: 10.88–22.43) of having catastrophic health expenditure compared to those in income decile ten (Table 3).

**Table 3:** Generalised Estimating Equation model of likelihood of having catastrophic healthcare expenditure

Parameter	Odds Ratio	95% Confidence Limits		*p*-value
Age (years)	1.04	1.04	1.04	<.0001
Income decile 1	15.63	10.88	22.43	<.0001
Income decile 2	7.43	5.74	9.61	<.0001
Income decile 3	6.12	4.90	7.65	<.0001
Income decile 4	4.43	3.71	5.28	<.0001
Income decile 5	4.81	3.98	5.82	<.0001
Income decile 6	2.38	2.18	2.59	<.0001
Income decile 7	2.58	2.33	2.85	<.0001
Income decile 8	1.79	1.71	1.87	0.0001
Income decile 9	1.67	1.62	1.72	0.0013
Income decile 10	REFERENCE			
Very poor self assessed health	1.24	1.23	1.26	0.1221
Poor self assessed health	1.35	1.34	1.37	0.0064
Fair self assessed health	1.25	1.25	1.26	0.0319
Good self assessed health	1.02	1.00	1.04	0.8705
Excellent self assessed health	REFERENCE			
2006	0.75	0.73	0.78	<.0001
2007	0.80	0.78	0.83	0.0062
2008	0.91	0.89	0.93	0.2041
2009	0.88	0.87	0.90	0.051
2010	0.88	0.87	0.89	0.0299
2011	0.96	0.95	0.97	0.4938
2012	1.01	1.00	1.01	0.8708
2013	1.00	1.00	1.00	0.9945
2014	REFERENCE			

Finally, we estimated the number of people who would have been classified as being in income poverty, had income been adjusted for the amount of healthcare expenditure. In 2006, an additional 141,000 people were in income poverty. In 2014, an additional 285,000 people were in income poverty (Table 4).

**Table 4:** Additional number of people who would be in income poverty when household income is adjusted for household healthcare expenditure

Year	Actual number of people in income poverty	Number of people in income poverty, adjusted for healthcare costs	Additional number of people in poverty
2006	1,187,000	1,328,000	141,000
2007	1,392,000	1,545,000	153,000
2008	1,371,000	1,524,000	153,000
2009	1,388,000	1,549,000	161,000
2010	1,424,000	1,639,000	215,000
2011	1,410,000	1,617,000	207,000
2012	1,436,000	1,658,000	222,000
2013	1,408,000	1,607,000	199,000
2014	1,302,000	1,587,000	285,000

## Discussion

Average household out-of-pocket expenditure on healthcare – covering expenditure on health practitioners, medication and private health insurance premiums – has remained relatively constant after adjusting for inflation between 2006 and 2014 for the general adult population in Australia. However, those with lower incomes were more likely to have catastrophic healthcare expenditures (spending 10% of more of household income on healthcare) over this time period, and between 2006 and 2014 there was increasing inequality in the distribution of catastrophic healthcare expenditure towards those with lower income. The impact of household healthcare expenditure on household living standards was such that after adjusting household income for healthcare expenditure in excess of 200,000 additional people would be classified as being in income poverty within Australia in 2014.

No previous study has sought to assess the distribution of the impact of out-of-pocket expenditure, nor sought to assess the impoverishing consequences of healthcare expenditure in Australia. Co-payments and the impact they have had on accessing primary health care services in Australia was discussed by Laba et al. [20], and a previous study has shown that 1 in 4 Australians with a chronic health condition skip care due to the cost [6]. This highlights the importance of assessing the level of out-of-pocket expenditure on healthcare and identifying population groups who may be disproportionately affected.

The use of self-reported healthcare expenditure is a key weakness of this study, which is also common to all previous studies that used individual-level data to assess of out-of-pocket health care expenditure in Australia. It could be questioned whether individuals are able to accurately recall the amount they have spent on healthcare, which may have influenced the accuracy of the results. However, the amount of expenditure reported in this study was similar to the amount reported in a previous study on healthcare expenditure [35]. Future research may be able to make better use of health administrative data to overcome these issues or use short recall periods [10].

Financial risk protection is a core objective of universal health coverage [33]. Although Australia has a universal health care system, this study has demonstrated that Australia’s health system may not be protecting its most vulnerable citizens against catastrophic health expenditure and income poverty, which disproportionally burdens the most disadvantaged people within the population. If a health care system is to meet the objectives of universal health coverage, total contributions should be based on ability to pay, and health care services should be allocated according to need, which means poorer people should receive greater health care benefits due to greater health care needs [33]. Through the Extended Medicare Safety Net Scheme the Australian government seeks to do this. However this study indicates that Australia’s universal health system appears to not safeguard the poorest people in society, which are the people who need it the most, against the financial hardship associated with accessing health care. Universal health systems should develop in a way that does not impose harm to other social sectors in people’s lives by imposing catastrophic health expenditures upon households.

## Conclusions

Out-of-pocket payments are considered to be the most regressive form of financing a health system [33]. These results highlight the financial impact experienced by households as a consequence of this regressive approach to providing health care to the population. The findings clearly demonstrate the importance of vigilance to ensure ongoing progress towards universal health coverage, rather than assuming that financial risk protection is an inevitable outcome of having a universal health system.

## Abbreviations

HIC: High Income Country: defined by the World Bank as a country with a gross national income per capita US$12,056 or more in 2017
HILDA: Household Income and Labour Dynamics in Australia: a longitudinal survey of private Australian households
OECD: Oganisation for Economic Co-operation Development: is an intergovernmental economic organisation with 36 member countries, founded in 1961 to stimulate economic progress and world trade
UHC: Universal Health Care: is a health care system that provides health care and financial protection to all citizens of a particular country
